# Reactivity to human papillomavirus type 16 L1 virus-like particles in sera from patients with genital cancer and patients with carcinomas at five different extragenital sites

**DOI:** 10.1038/sj.bjc.6600870

**Published:** 2003-04-01

**Authors:** G J J Van Doornum, C M Korse, J C G M Buning-Kager, J M G Bonfrer, S Horenblas, B G Taal, J Dillner

**Affiliations:** 1 Slotervaart Hospital, Amsterdam, The Netherlands; 2 Antoni van Leeuwenhoek Hospital and Netherlands Cancer Institute, Amsterdam, The Netherlands; 3 Microbiology and Tumor Biology Centre, Karolinska Institute, Stockholm, Sweden

**Keywords:** HPV 16 serology, oropharyngeal carcinoma, oesophageal carcinoma, cervical cancer, vaginal and vulvar cancer, penile carcinoma

## Abstract

A retrospective seroepidemiologic study was performed to examine the association between human papillomaviruses (HPV) 16 infection and carcinomas of the oropharynx, the oesophagus, penis and vagina. Sera were selected from the serum bank from the Antoni van Leeuwenhoek Hospital (Netherlands Cancer Institute) and the Slotervaart Hospital in Amsterdam, the Netherlands. Presence of HPV 16 specific antibody was assessed using HPV 16 L1 capsids. Sera positive for HPV 16 capsid antibody were further tested for antibody against HPV 16 E7 peptides. Prevalence of antibody against HPV 16 L1 capsids among both the negative control group without cancer and the negative control group with gastric cancer was 18%, while seroprevalence among the control group of patients with HPV-associated cervical squamous cell carcinoma was 47% (*P*<0.001). Among the patients with penile squamous cell carcinoma seroprevalence was 38% (*P*<0.001), among patients with oropharyngeal carcinoma 33% (*P*=0.04) and among patients with oesophageal squamous cell carcinoma 14% (*P*=0.7). The serological evidence for association between HPV 16 infection and both oropharyngeal carcinoma and penile carcinoma was established. The conclusion that no association was found between the presence of antibody against HPV 16 L1 capsids and oesophageal squamous cell carcinoma was in accordance with results of other studies carried out in the Netherlands using HPV DNA technology. In the subjects with HPV 16 L1 capsid antibody, no association was found between the antibody against HPV 16 E7 and clinical outcome.

The causative association between infection with oncogenic human papillomaviruses (HPV) and cervical squamous cell carcinoma has been established by molecular and epidemiological studies (Muñoz and Bosch, 1989; zur Hausen, 1994). Current infection by HPV can be detected by polymerase chain reaction (PCR) or hybrization techniques. Human papillomavirus serology can be applied to determine past or present HPV infection (de Villiers, 1992; Kirnbauer *et al*, 1994). Serological assays based on HPV capsids, which represent conformational viral epitopes, can detect HPV-type restricted antibodies. These assays have been validated as useful tools for seroepidemiologic studies among various cohorts (Van Doornum *et al*, 1994,^1998^; Dillner, 1995; Nonnenmacher *et al*, 1995,^1996^; Wideroff *et al*, 1995,1996a; Wikström *et al*, 1995a,1995b; Lehtinen *et al*, 1996).

Results of some studies suggest that HPV infection may be a risk factor for squamous cell carcinoma of the head and neck or encompasses a distinct entity with an increased sensitivity to radiotherapy (Gillison *et al*, 2000; Forastiere *et al*, 2001; Lindel *et al*, 2001; Mork *et al*, 2001). Squamous cell oesophageal cancer shows wide regional variation in incidence and causal association (Sur and Cooper, 1998). With regard to the association between HPV infection and oesophageal squamous cell carcinoma, contradictory molecular and serological findings are reported (Dillner *et al*, 1995; Bjørge *et al*, 1997b; Kok *et al*, 1997; Miller *et al*, 1997; Saegusa *et al*, 1997; de Villiers *et al*, 1999; Lagergren *et al*, 1999; Lavergne *et al*, 1999). For the Netherlands, that are reported to be a low-risk area for squamous cell cancer of the oesophagus, using HPV DNA technology no evidence was found for a role of HPV as a causative agent (Kok *et al*, 1997). However, while detection of HPV DNA determines only current infection, an advantage of serological studies is that they can give insight into exposure to papillomaviruses in the past.

Presence of HPV DNA in carcinomas of the penis is observed in a proportion of the specimens analysed (Grussendorf-Cronen, 1997; Tornesello *et al*, 1997; Dianzani *et al*, 1998; Levi *et al*, 1998; Poblet *et al*, 1999; Dillner *et al*, 2000; Rubin *et al*, 2001). Some serological studies relative to HPV infection in individuals with penile carcinoma were carried out in relatively small groups of patients (Wideroff *et al*, 1996b; Strickler *et al*, 1998; Carter *et al*, 2001).

With regard to vaginal and vulvar cancer, little is known about the prevalence of antibody against HPV among patients with these cancers, although HPV infection is considered as a risk factor for vaginal cancer (Daling *et al*, 2002).

The objective of this retrospective study was to determine the seroprevalence of antibody against HPV 16 viral-like particles (VLP) containing L1 proteins among Dutch patients with carcinomas in the oropharyngeal region, tongue and larynx, patients with oesophageal carcinoma, women with vaginal carcinoma and men with penile carcinoma. Sera positive for antibody against HPV 16 L1 were tested for the presence of antibody to E7.

## MATERIALS AND METHODS

Serum specimens were taken from the serum bank at the Antoni van Leeuwenhoek (AvL) Hospital and Netherlands Cancer Institute in Amsterdam, the Netherlands. Cases were selected over a period of 10 years, 1989–1999, based on the diagnosis as stated in the anonymised medical records. The inclusion criterion was the site of the cancer: penis, cervix, vagina, the upper aerodigestive tract (larynx, oropharynx, tongue), oesophagus and stomach. The anatomical sites were defined according to the codes of the International Classification of Diseases (WHO, 1979). Sera from patients with cervical squamous cell carcinoma were considered as HPV-positive cancer control sera. Sera from patients with gastric carcinoma were considered as HPV-negative cancer control sera. For the HPV-negative control group without cancer, serum specimens were selected from the serum bank in the Slotervaart (SL) hospital. These serum specimens were obtained from patients referred to the gastroenterology clinic for gastric ulcer complaints during 1999. The control group was matched on gender and age with the gastric carcinoma cases.

The sera from the AvL patients were obtained at the first visit to the hospital during the period wherein the diagnosis was made. The sera from the SL hospital patients were also taken at the first visit to the gastroenterology clinic. The specimens were stored at −20°C and transported on dry ice to the Karolinska Institute, where the assays were performed by CM Korse and JCGM Buning-Kager.

The pathological diagnosis was made at the Department of Pathology of the AvL hospital and the SL hospital, respectively, using standard methods, and coded according to international standards (WHO, 1979).

First, all sera were screened on the presence of IgG antibody against HPV 16 capsids containing L1 proteins. The positive sera were further tested on the presence of IgG antibody against HPV 16 E7 peptides.

### Enzyme-linked immunosorbent assays

Baculovirus-expressed HPV16 capsids containing both the L1 proteins were diluted in cold phosphate-buffered solution (PBS) to 1 *μ*g ml^−1^, coated overnight at 4°C onto microtiter plates and subsequently blocked with 10% horse serum in PBS (HS-PBS). Sera were diluted 1 : 30 with HS-PBS and added to the plates for 2 h at room temperature (RT). A mouse monoclonal antibody to human IgG, diluted 1 : 800, was added and incubated for 90 min at room temperature, followed by a horseradish peroxidase-conjugated antibody to mouse IgG. Before every addition, the plates had been washed with PBS-0.1% Tween. For each sample the absorbency of the same sample obtained from a well coated with disrupted bovine papilloma virus (BPV) was subtracted from the absorbency obtained for the corresponding well coated with HPV 16 capsids.

The assays included two internal positive and one negative control specimens on each ELISA plate. Three pools of sera from patients with cervical carcinoma, from patients with cervical intraepithelial neoplasia (CIN) and from healthy blood donors were used for this purpose. The absorbencies read from each plate were normalised relative to the results of the internal standard. The internal standard was measured in a plate with negative control sera obtained from sexually inexperienced women. Cutoff level was defined as the mean value of the absorbencies of the plate with the negative controls plus three standard deviations.

For the measurement of E7 peptides the same protocol was used. The only difference was that the peptide was diluted to 1 mg ml^−1^ in Tris buffer and coated overnight at room temperature. As negative control another plate was coated with Tris buffer only. The E7 peptide has the designation E7-2 and has the amino-acid sequence PETTDLYCYEQLNDSSEEED (Dillner, 1990).

### Data analysis

The data were analysed using the Statistical Package for Social Science (SPSS/PC Version 10.0.5) and Epi Info Version 6.0 (Dean *et al*, 1994). To compare the proportions the *χ*^2^ method was used, and Fisher's exact test when appropriate. A two-tailed *P*-value of less than 0.05 was considered to indicate statistical significance.

## RESULTS

The characteristics such as gender and age of the patients from whom the serum specimens were obtained are presented according to pathological diagnosis in Table 1

**Table 1:** Characteristics of 606 patients with various carcinomas and 100 patients selected as negative control group

	***N***	**Mean age (years)**	**Male**	**Mean age (male)**	**Female**	**Mean age (female)**
Negative control (no cancer)	100	61.8	50	61.3	50	62.4
Negative control (stomach carcinoma)	54	61.5	37	61.2	17	62.3
Cervical squamous carcinoma	51	63.7			51	63.7
Tongue carcinoma	56	58.7	30	59.1	26	58.3
Oropharyngeal carcinoma	48	56.1	33	57.2	15	53.8
Oesophageal carcinoma	117	64.5	86	63.8	31	66.5
Squamous cell carcinoma	56	64.9	34	64.6	22	65.5
Adenocarcinoma	48	64.4	41	63.1	7	71.6
Others	13	63.2	11	63.7	2	60.6
Laryngeal carcinoma	127	62.1	111	62.7	16	58.0
Vaginal carcinoma	31	71.5			31	71.5
Penile carcinoma	122	60.9	122	60.9		
						
Total	706	61.9	417	61.3	228	62.9

. The seroprevalence of antibody to HPV 16 L1 capsids among the various groups is shown in Table 2

**Table 2:** Results of antibody testing against HPV 16 L1 capsids in 606 patients with various carcinomas and results of antibody testing against HPV 16 E7 peptides assay carried out in the HPV 16 L1 capsid antibody positive samples

	***N***	**HPV 16 pos Pos/*N* (%)**	***P*-value**^a^	**OR**^b^	**E7 Pos/*N* (%)**
Negative control (no cancer)	100	18/100 (18)			2/18 (11)
Negative control (stomach carcinoma)	54	10/54 (18)			0/9 (0)
Cervical squamous carcinoma	51	24/51 (47)	<0.001	4.00	7/24 (29)
				(1.9–8.4)	
Tongue carcinoma	56	12/56 (21)	0.741		3/12 (25)
Oropharyngeal carcinoma	48	16/48 (33)	0.043	2.25	2/16 (12)
				(1.0–4.9)	
Laryngeal carcinoma	127	25/127 (20)	0.876		3/25 (12)
Oesophageal carcinoma	117	20/117(17)			0/20 (0)
Squamous cell	56	8/56 (14)	0.649		
Adenocarcinoma	48	11/48 (23)			
Others	13	1/13 (8)			
					
Vaginal carcinoma	31	8/31 (26)	0.466		0/8 (0)
Penile carcinoma	122	46/122 (38)	<0.001	2.72	5/45 (11)
				(1.5–4.9)	
Total	706	align="center"179/706(25)			22/177(12)

a Yates corrected.

b Odds ratio (95% confidence limits).

. The sera positive for antibody against HPV 16 L1 capsid were further tested for the presence of antibody to HPV 16 E7 peptides; the results are also shown in Table 2. The seroprevalence found in the negative control groups was 18% both in the stomach cancer control group (10 out of 54) and in the noncancer gastric ulcer group (18 out of 100). The seroprevalence in the cervical squamous cell cancer group was 47% (24 out of 51); the difference with the negative control group was statistically significant (*P*<0.001). A total of 46 of the 122 (38%) individuals with penile cancer were positive for HPV 16 L1 antibody (*P*<0.001). The proportion of individuals with oesophageal squamous cell carcinoma with antibody against HPV 16 L1 capsids was only 14% (8/56), whereas 11 of the 48 (23%) sera from patients with oesophageal adenocarcinoma were positive for HPV 16 L1 antibody. In the group of patients with oropharyngeal carcinoma, 16 out of 48 (33%) individuals were positive for antibody against HPV 16 L1 antibody (*P*=0.04). The seroprevalence found in the patients with tongue carcinoma or laryngeal cancer was 12 out of 56 (21%) and 25 out of 127 (20%), respectively. A total of 8 out of 31 (26%) of the patients with vaginal carcinoma were positive (*P*=0.47). In the negative control sera that were positive for HPV 16 L1 antibody, two of 27 (7%) sera were also positive for antibody against HPV 16 E7 peptides, and seven of the 24 (29%) of the sera from patients with cervical squamous cell carcinoma, who were considered as positive control group (*P*=0.07, Fisher's exact two-tailed). No statistically significant difference was observed for the presence of HPV 16 E7 antibody in any of the other groups studied.

In the group of patients with cervical carcinoma and penile carcinoma, the possible association between clinical outcome and presence of antibody both against L1 capsids and E7 peptide was analysed. As clinical outcome the following categories were chosen: alive with or without tumour and died with or without tumour. For 21 of the 24 subjects with cervical carcinoma, who were positive for HPV 16 L1 antibodies and in whose serum samples antibody against E7 was determined, the follow-up data were available. There were no statistical differences between the subjects with or without antibodies against E7 with regard to survival (*P*=0.21, Fisher's exact test, two-sided). The same analysis was executed for the subjects with penile carcinoma, again no differences could be found (*P*=1.0, Fisher's exact test, two-sided).

## DISCUSSION

In this study, we analysed the prevalence of HPV 16 specific antibody in patients with a variety of cancers. As HPV seropositive cancer control subjects were included patients with cervical squamous cell carcinoma, and patients with gastric ulcer or stomach carcinoma as HPV negative control patients without and with cancer, respectively.

We realise that a retrospective, seroepidemiological study using hospital-based negative control subjects has some limitations in comparison with a nationwide seroepidemiological case–control study or prospective studies as carried out in the Nordic countries to study the association between oesophageal carcinoma and HPV antibody positivity (Dillner *et al*, 1995; Bjørge *et al*, 1997a,1997b; Lagergren *et al*, 1999). Nevertheless, results of a retrospective case–control study can be used to determine disease aetiology. Furthermore, the number of case subjects in the various subgroups in our study was relatively large. A problem arises in the selection of the control subjects, as these were taken from the hospital population on practical considerations. Therefore, we included a negative control group without cancer as well as a control group with cancer that is not considered to be caused by an HPV infection.

The seroprevalence of 18% for HPV 16 antibody in the HPV-negative control groups in our study seemed rather high, but could be considered consistent with figures reported previously from The Netherlands. In Dutch patients who were HPV DNA negative and participated in Amsterdam, The Netherlands, in a nonintervention study relative to cervical dysplasia a seroprevalence of 14.6% was reported (Bontkes *et al*, 1999). In studies on HPV seroprevalence among heterosexuals with multiple partners, we found previously a frequency of 23 and 20% of IgG antibody against HPV 16 L1 peptides and HPV 16 L1 VLP, respectively (Van Doornum *et al*, 1994,^1998^).

Seroprevalences must be compared with local or regional figures as the values can differ in different groups of subjects living in various countries. In a study described by Wideroff *et al* (1999) seroprevalence for HPV 16 IgG antibody among cases with squamous intraepithelial lesions (SIL) was 51.3% and for the random control group the seroprevalence for HPV 16 was 15.9%. On the other hand, among high-risk populations in Greenland and Denmark, 56.2% of the Greenlander women and 41.1% of the Danish women were found positive for antibody to HPV 16 (Nonnenmacher *et al*, 1996). And in another study carried out in South Africa among San people, also known as Bushmen, seroreactivity to HPV 16 was 16.1% among adults (Marais *et al*, 2000).

### Oropharyngeal carcinoma

The seroprevalence for patients with oropharyngeal cancer of 33% (16 out of 48) in the present study is comparable with the prevalence of 38% (10 out of 26) *vs* 10% (14 out of 137) among control subjects reported in a recently published nested case–control study among patients with head and neck cancers in the Nordic countries (Mork *et al*, 2001). In that study a significant relation was also found between seropositivity for HPV 16 antibody and cancers originating from mucosal stratified squamous cell epithelium in the head and neck region. In another study, the authors suggested a causative association between HPV and a subset of squamous cell cancers of head and neck, more case–control studies were considered to be needed to assess that interaction (Franceschi *et al*, 2000; Gillison *et al*, 2000).

### Tongue and laryngeal carcinomas

Although the prevalence of 21% for positivity of HPV 16 antibody among patients with tongue cancer found in the present study was greater than the 16% found in the study of Mork, we could not conclude that there was an association between presence of HPV antibody and tongue cancer because the proportion of HPV 16 seropositive control subjects was higher in the present study than among the control patients studied by Mork, 18 *vs* 7% (Mork *et al*, 2001). The same goes for the figures found for the HPV 16 seroprevalence among the patients with laryngeal cancers.

### Oesophageal carcinoma

In the present study conducted among Dutch patients, we did not find an association between positivity for HPV 16 antibody and oesophageal carcinoma. This is in accordance with other studies as reported by Lagergren *et al* (1999). In the past, prospective seroepidemiological evidence had been brought forward that HPV 16 infection might be a risk factor for oesophageal carcinoma in Norway and Finland (Dillner *et al*, 1995; Bjørge *et al*, 1997a,1997b). Contrary to these findings, the seroepidemiologic case–control study later performed by Lagergren *et al* (1999) in Sweden could not support the conclusions from the above-mentioned studies. It must be noted that in various studies, the seroprevalence among the control subjects differ. For example, in the study in which Bjørge reported an association between HPV 16 seropositivity and oesophageal cancer, the seroprevalence for HPV 16 in patients with oesophageal squamous cell carcinoma was 12 *vs* 5% in the controls. In the study reported by Dillner (1995) among Finnish patients with oesophageal carcinoma, the seroprevalence of antibody against HPV 16 was 21 *vs* 3% among matched controls. In the study of Lagergren, seroprevalence of HPV 16 antibody was 11.6% in the case subjects with oesophageal squamous cell carcinoma, and 10.9% in the control subjects, while in the present study, the seroprevalence among cases with oesophageal carcinoma was 14 *vs* 18% among the negative control group.

It must be also taken into account that the role of HPV infection in squamous cell oesophageal cancer shows geographic variation (Sur and Cooper, 1998). This variation may be because of the fact that oesophageal carcinogenesis is a complex multistep process. For instance, in a study among Japanese patients, it was concluded that HPV was not likely to be involved in oesophageal squamous cell carcinoma, and in only 10 of 22 Alaska native patients with squamous cell carcinoma of the oesophagus HPV DNA was detected (Miller *et al*, 1997; Saegusa *et al*, 1997). In Chinese patients originating from a high-incidence area, HPV DNA was demonstrated in 20 out of 117 (17%) of the cases (De Villiers *et al*, 1999). However, only 3 out of 117 (3%) was of the high-risk HPV type. In a study performed in The Netherlands, no evidence could be found for a role of HPV in squamous cell carcinoma of the oesophagus (Smits *et al*, 1995; Kok *et al*, 1997). In two other West European studies performed in Belgium and France, the role of HPV infection in the pathogenesis of oesophageal squamous cell carcinoma could also not be demonstrated (Benamouzig *et al*, 1995; Lambot *et al*, 2000).

The suggestion was put forward that there might be a difference between the results from studies executed in high-risk and low-risk areas for oesophageal carcinoma because of the differing time of diagnosis of the cancer. Another explanation might be that the tissue of an early diagnosed oesophageal carcinoma or premalignant papilloma might contain HPV DNA and/or HPV antigens, and not the specimens obtained from advanced oesophageal carcinoma. This is by analogy with bovine oesophageal carcinoma and the association with bovine papillomavirus (BPV) type 4. BPV 4 DNA and antigens can be found in high copy numbers in the premalignant oesophageal papillomas, but not in the carcinomas. Cattle infected with BPV 4, which feed on bracken fern, have a high risk of oesophageal cancer, but the BPV 4 viral genome is not present in frank cancers or fully transformed cells (Campo, 1992). If this phenomenon holds true for human oesophageal carcinoma, we should predict a high seroprevalence for antibody against HPV. In fact, we did not find a greater prevalence of antibody against HPV 16 capsids among patients with oesophageal cancer than in control patients.

### Penile carcinoma

Only a few studies are reported on penile cancer and the role of HPV; a review is given by Griffiths and Mellon (1999) and later by Dillner *et al* (2000). The prevalence of subclinical or latent penile HPV infections among young, sexually active and healthy individuals is reported between 20 and 50%, while the incidence of penile carcinoma is relatively low (Van Doornum *et al*, 1994; Wikström *et al*, 2000). In penile carcinoma patients from Argentina, HPV DNA was detected in 27 out of 38 (71%) of the cases using single-stranded conformational polymorphism; 10 of the 38 (26.3%) patients were found positive for HPV 16 (Picconi *et al*, 2000). The difference with the prevalence of HPV 16 specific antibody found in the present study might be attributed to geographical distribution or a specific subtype of penile cancer. In a study on formalin-embedded tissue samples collected from the USA and Paraguay using the sensitive SPF 10 primers HPV DNA was detected in 80% of basaloid and 100% of warty tumour subtypes, whereas keratinising squamous cell carcinomas and verruccous carcinomas were positive for HPV DNA in 34.9 and 33.3% of the cases, respectively (Rubin *et al*, 2001). With regard to antibody against HPV, one study on the seroprevalence of HPV 16 antibody among Chinese patients with penile cancer reported that no antibodies against HPV 16 VLPs were detected in serum specimens of 55 Chinese patients with penile cancer, nor in the sera of 60 controls (Wideroff *et al*, 1996b). In an HPV 16 serosurvey of cancer patients, a small subset of eight patients with penile squamous carcinoma was described with five of them positive for HPV 16 antibody (Strickler *et al*, 1998). In a study from Seattle, a seroprevalence of HPV 16 antibody for *in situ* penile cancer of 20.3% was found, and 28.5% for invasive penile cancer (Carter *et al*, 2001). Thus, the findings of our study are in accordance with the latter studies indicating a relation between HPV infection and penile carcinoma.

### Vaginal carcinoma

The elevated HPV 16 seroprevalence among patients with vaginal cancer was not statistically significant compared to the control women. This is in contrast to the results of a recently published report that antibodies to HPV 16 L1 were strongly related to the risk of vaginal cancer (Daling *et al*, 2002). Approximately 44% of both the *in situ* and the invasive case subjects had antibodies to HPV 16 L1 compared to 15% of the control women.

### Antibody against E7

Presence of antibodies against papillomavirus might be used as prognostic marker in cervical cancer patients (Heim *et al*, 2002). If expression of transforming proteins as E6 and E7 is implicated in the carcinogenesis of epithelial carcinoma, it might be hypothesised that development of antibody against either of these oncoproteins is related with clinical outcome. Antibody responses against E6 and E7 proteins have been shown to be associated with clinical stage of cervical carcinoma (Zumbach *et al*, 2002). In control groups of that study a prevalence of 2% was found, whereas 26 of 95 (27%) patients with cervical carcinoma showed antibody against HPV 16 E6 or E7. Antibody against HPV 16 E6 was dominant among patients with HPV 16 specific antibody (23 out of 27, 85%) over that to E7 (9 out of 27, 33%). E6 and/or E7 antibody prevalence increased from 21% in FIGO stage-I patients to 42% in stage-II patients and reached 52% in stage-III patients. However, in the present study, among patients with cervical carcinoma who were HPV 16 L1 antibody positive, a prevalence of 7 out of 24 (29%) was found for antibody against HPV 16 E7 peptides. Analysis of the association between the presence of E7 antibody and the clinical outcome yielded no significant result, indicating that E7 antibody does not improve clinical outcome. This finding is in accordance with the conclusion drawn in a study from Sweden, in which antibodies to HPV 16 capsids and to the oncoproteins E6 and E7 did not appear to be prognostic indicators of cervical cancer prognosis (Sillins *et al*, 2002).

In conclusion, we found evidence for a strong association between the presence of HPV 16 L1 antibody and both cervical squamous cell carcinoma and penile squamous cell carcinoma. A relation between the presence of HPV 16 antibody and oropharyngeal cancer was also found. No serological evidence was demonstrated for an association between HPV 16 antibody and oesophageal, tongue, laryngeal and vaginal carcinoma.
